# Combined analysis of endometrial thickness and pattern in predicting outcome of in vitro fertilization and embryo transfer: a retrospective cohort study

**DOI:** 10.1186/1477-7827-8-30

**Published:** 2010-03-24

**Authors:** Shi-Ling Chen, Fang-Rong Wu, Chen Luo, Xin Chen, Xiao-Yun Shi, Hai-Yan Zheng, Yun-Ping Ni

**Affiliations:** 1 Center for Reproductive Medicine, Department of Obstetrics and Gynecology, Nanfang Hospital, Southern Medical University, Guangzhou, People's Republic of China

## Abstract

**Objective:**

To evaluate the combined effect of endometrial thickness and pattern on clinical outcome in patients undergoing in vitro fertilization/intracytoplasmic sperm injection and embryo transfer (IVF/ICSI-ET).

**Methods:**

Cycles of IVF/ICSI-ET conducted between January 2003 and December 2008 at a university-based reproductive center were reviewed retrospectively. Endometrial ultrasonographic characteristics were recorded on the day of hCG administration. In the combined analysis, endometrial thickness groups (group 1: equal or <7 mm; group 2: 7-14 mm; group 3: >14 mm) were subdivided into two endometrial patterns (pattern A: triple-line; pattern B: no-triple line). Clinical pregnancy rate (CPR) and early miscarriage rate in different groups were analyzed.

**Results:**

A total of 2896 cycles were reviewed. Clinical pregnancy rate (CPR) was 24.4% in group1-A. There were no second trimester pregnancies in group 1-B. Miscarriage rate in group 2-A was significantly lower compared to group 2-B (P < 0.01), although CPR did not show any significant differences between the groups. A no-triple line endometrial pattern with moderate endometrial thickness (7-14 mm) had a detrimental effect on pregnancy outcome, but not the occurrence of pregnancy. In group 3, there was no difference in CPR and miscarriage rates between the two patterns; adequate endometrial thickness (>14 mm) seemed to mitigate the detrimental impact (high miscarriage rate) of pattern B.

**Conclusion:**

Combined analysis of endometrial thickness and pattern on the day of hCG administration was a better predictor of the outcome of IVF/ICSI-ET and may be more helpful for patient counseling than the separate analyses.

## Background

It is generally accepted that endometrial receptivity is critical to successful pregnancy. Ultrasonographic examination has been routinely performed in assisted reproduction technology (ART) treatments because of the accurate evaluation and noninvasive detection. Indeed, both endometrial thickness and endometrial pattern have been regarded as prognostic parameters for successful pregnancy in in vitro fertilization/intracytoplasmic sperm injection and embryo transfer (IVF/ICSI-ET).

Following the periodic stimulation of ovarian hormones, the changes in endometrial structure during the menstrual cycle can be identified easily by ultrasound examination [1]. In an IVF/ICSI procedure, hCG is used as a substitute for the natural luteinizing hormone (LH)-surge to trigger the final maturity of the oocyte. Evaluation of endomtrium on the day of hCG administration is of great clinical importance [2,3]. Several studies have demonstrated the existence of a correlation between endometrial characteristics and pregnancy rate in IVF/ICSI patients [4-6]. However, the correlation proposed in these studies has not been universally accepted [7,8]. There also is no consensus on whether the endometrial ultrasound characteristics can predict the pregnancy outcome in IVF/ICSI treatment. Weissman *et al. *reported an increase in miscarriage rate when an endometrial thickness of > 14 mm was found on the day of hCG injection [9]. However, data from more recent studies do not support this finding [10]. A no triple-line endometrial pattern seems to be a prognostic sign of a less favourable outcome, while a triple-line pattern appear to be associated with conception [11-13].

Despite the abundance of studies that have evaluated the effect of endometrial characteristics on clinical outcome in IVF/ICSI patients, studies on the combined predictive role of endometrial thickness and pattern are lacking. In this study, we examined the correlation between endometrial thickness and pattern (individually and together) and IVF/ICSI outcome. Our objective was to investigate whether combined analysis of endometrial thickness and pattern would improve the prediction of clinical outcome compared to the analysis of thickness or pattern separately.

## Methods

### Study population

Cycles of IVF/ICSI conducted between January 2003 and December 2008 at a university-based reproductive center were reviewed retrospectively. Since this study was retrospective and vaginal sonographic assessment on the day of hCG administration was done routinely in our center, Institutional Review Board approval for the study was not necessary. All fresh IVF or ICSI treatment cycles that used the long protocol (midluteal phase GnRH-agonist suppression) as the method of ovarian stimulation and reached oocyte pick up and embryo transfer within the study period were included, regardless of diagnosis, reproductive history, or insemination method. Cycles using donor oocytes or cryopreserved embryos were excluded from this study. Other exclusion criteria included: age greater than 42 years, the presence of known endometrial anomalies, and ovarian stimulation method other than the long protocol.

### IVF/ICSI-ET treatment protocol

For ovarian stimulation utilizing the long protocol procedure, each patient received a single intramuscular (IM) injection of the gonadotrophin releasing hormone (GnRH) agonist (1.25 mg to 1.875 mg) in the midluteal phase of the cycle prior to the initiation of controlled ovarian hyperstimulation (COH). After spontaneous menstruation occurred, a vaginal ultrasound examination and serum estradiol concentration (E2) measurement were performed. When the E2 levels were ≤50 pg/mL, and the longest follicle diameter was <10 mm without ovarian cysts, COH was performed. COH was achieved with administration of gonadotrophin, including the follicle stimulating hormones (FSH) and/or human menopausal gonadotrophin (hMG). The initial dosage of gonadotrophin ranged from 150 to 450 IU, depending on the basal FSH level, antral follicular count (AFC), and maternal age. Once three or more follicles reached a diameter of ≥17 mm, hCG was administered. Oocytes were retrieved within 34 to 36 hours and embryo transfer was performed 2 to 5 days afterwards. All patients were given IM P daily starting on the day of oocyte retrieval. Serum β-hCG levels were measured 11-14 days after embryos transfer. Subsequent ultrasound examinations were performed at a gestational age of 7 weeks. Clinical pregnancy was defined as identification of a gestational sac 2-3 weeks after embryo transfer. Early miscarriage was defined as pregnancy ending before 12 weeks of gestation.

### Ultrasound examinations

**Figure 1:**
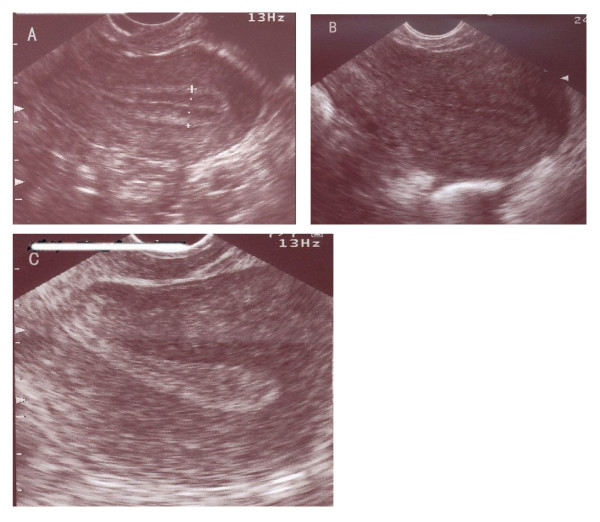
**Longitudinal ultrasound images demonstrate the endometrial pattern**. (A) Triple-line pattern: hypoechoic endometrium with well-defined hyperechoic outer walls and a central echogenic line; (B) No-triple line pattern: isoechoic endometrium with poorly defined outer walls and central echogenic line; (C) No-triple line pattern: homogeneous hyperechoic endometrium.

Endometrial thickness was measured in the midsagittal plane of the uterine body on the day of hCG administration. The largest thickness from one interface of the endometrial-myometrial junction to the other was measured. All cycles were divided into three groups depending on the thickness: group 1 ≤7 mm; group 2: >7 to ≤14 mm; group 3: >14 mm. Endometrial pattern is defined as the type of relative echogenicity of the endometrium compared with adjacent myometrium [2]. Several classifications of ultrasonographic endometrial patterns have been used in the previous studies. In our study, we classified all patterns into two types. Pattern A (triple-line) was described as hypoechoic endometrium with well-defined hyperechoic outer walls and a central echogenic line; pattern B (no triple-line) was defined as a isoechoic or homogeneous hyperechoic endometrium with a non-prominent or absent central echogenic line (Figure 1).

### Statistical analysis

Data were analyzed using SPSS version 13 software (SPSS Inc., Chicago, IL), all tests were two-tailed, and P < 0.05 was considered statistically significant. Continuous variables were presented as mean ± SD and were tested by student's t-test. Categorical data were expressed as numbers and compared using the Chi-square test. Logistic regression was performed to determine the independent effect of individual variables on clinical outcome.

## Results

### Baseline cycle characteristics

A total of 2896 IVF/ICSI cycles were investigated in this study. The overall clinical pregnancy rate was 48.4% and the early miscarriage rate was 8.4%. Patients ranged in age from 20 to 42 years, and endometrial thickness on the day of hCG administration ranged from 5.2 mm to 26.7 mm. Other demographic data, such as antral follicular count (AFC), duration of infertility, and number of embryos transferred are summarized in Table 1.

**Table 1:** Baseline cycle characteristics (N = 2896)

Variable	Mean ± SD^a^
Maternal age (years)	31.0 ± 3.9
Duration of infertility (years)	5.0 ± 3.0
Baseline FSH (IU/L)	7.1 ± 2.2
AFC	15.6 ± 6.8
Duration of stimulation (days)	11.7 ± 3.4
Total dose of gonadotrophin (IU)	2537.3 ± 1067.2
Endometrial thickness (mm)	11.8 ± 2.7
E_2_on hCG day (pg/mL)	2107.3 ± 1596.1
P on hCG day (ng/mL)	1.0 ± 1.3
No. of oocyte retrieved	12.7 ± 6.1
No. of embryos transferred	2.3 ± 0.5
Etiology of infertility	
Tubal factor	55.8%
Ovulatory dysfunction	1.4%
Endometriosis	3.2%
Uterine factor	0.3%
Male factor	13.4%
Unknown factor and others	2.2%
Multiple factors	23.7%

^a^Mean ± SD, unless otherwise indicated

AFC = antral follicular count

P = serum progesterone concentration

E_2 _= serum estradiol concentration

### Binary logistic analysis of some variables on clinical pregnancy rate

Forward stepwise binary logistic regression analysis (model R^2 ^= .028, P < .001) was used to assess the effect of maternal age, AFC, endometrial thickness and pattern, length of infertility, P levels on the day of hCG administration, and basal FSH levels on clinical pregnancy. The analysis indicated that maternal age (r = -.025, P = .037) was negatively correlated with clinical pregnancy, and that increasing endometrial thickness (r = .060, P = .001) and AFC (r = .027, P < .001) were associated with improved clinical pregnancy rates. Other variables involved did not contribute significantly to clinical pregnancy (Table 2).

**Table 2:** Binary logistic regression^a ^(model R^2 ^= .028, *P *< .001)

Independent variables	R^b^	*P*^c^
Maternal age	-0.025	0.037
Endometrial thickness	0.060	0.001
AFC	0.027	0.000
Endometrial thickness	0.777	0.378
Baseline FSH level	0.087	0.768
P on hCG day	3.000	0.083
Duration of infertility	0.885	0.347

^**a**^Clinical pregnancy served as dependent variable; Method = forward stepwise (likelihood ratio)

^**b**^Partial coefficients

^**c**^*P *values. R values were considered statistically non-significant unless *P *< 0.05

### Endometrial pattern

**Figure 2:**
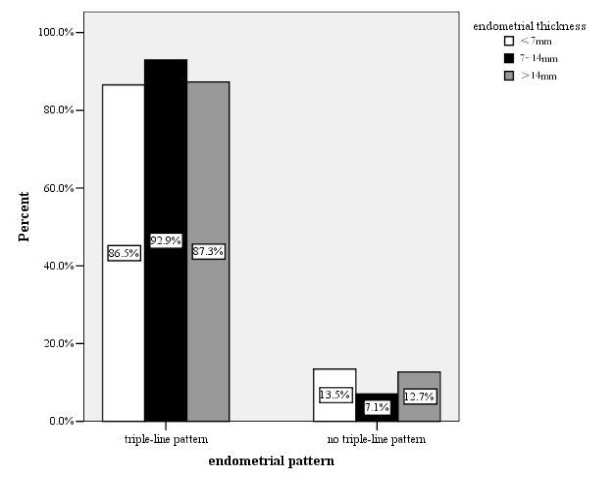
**Distribution of endometrial patterns in three endometrial thickness groups**. The percentages of endometrial pattern are shown for individual endometrial thickness group.

Most (91.9%) of our subjects had a triple-line pattern on the day of hCG administration. The distribution of endometrial patterns in the three endometrial thickness groups is shown in Figure 2. Pattern A was detected more frequently in group 2 than in groups 1 and 3(P < 0.01). Progesterone levels on the day of hCG administration did not differ between the two patterns (0.95 ± 0.82 in pattern A vs 1.04 ± 1.07 in pattern B, P > 0.05). There was no difference in clinical pregnancy rates between the patterns (48.6% in pattern A, and 46.2% in pattern B, P > 0.05). The incidence of miscarriage in pattern B was significant higher than that in pattern A (15.6% vs 7.9%, P < 0.05).

### Endometrial thickness

To evaluate the role of endometrial thickness in pregnancy outcome, clinical pregnancy rates were evaluated at each millimeter of endometrial thickness. Pregnancy rates ranged from 11.1% among patients with an endometrial thickness of ≤ 6 mm to 59.1% among patients with an endometrial thickness of 14-15 mm. Early miscarriage rates varied with endometrial thickness, but showed no consistent increase with increasing endometrial thickness (Table 3). An endometrial thickness threshold of 7 mm was observed below which pregnancy rates decreased rapidly. Clinical pregnancy rates were 23.1% in group 1 (≤ 7 mm), 47.6% in group 2 (7.1-14 mm) and 55.2% in group 3 (>14 mm), the differences between groups being statistically significant (P < 0.01). Miscarriage rates were not significantly different in the three groups (Table 4).

**Table 3:** Clinical outcome by individual endometrial thickness

Endometrial thickness (mm)	Cycles (n)	Clinical pregnancy rate (cycles, %)	Miscarriage rate (n, %)
≤ 6	9	1 (11.1)	0
>6 to ≤ 7	43	11 (25.6)	1 (9.1)
>7 to ≤ 8	142	64 (45.1)	4 (6.3)
>8 to ≤ 9	277	106 (38.3)	13 (12.3)
>9 to ≤ 10	393	191 (48.6)	16 (8.4)
>10 to ≤ 11	424	204 (48.1)	20 (9.8)
>11 to ≤ 12	416	198 (47.6)	14 (7.1)
>12 to ≤ 13	377	185 (49.1)	15 (8.1)
>13 to ≤ 14	320	170 (53.1)	17 (10.0)
>14 to ≤ 15	198	117 (59.1)	9 (7.7)
>15 to ≤ 16	124	57 (46.0)	3 (5.3)
>16 to ≤ 17	71	40 (56.3)	5 (12.5)
>17 to 26.7	102	59 (57.8)	1 (1.7)

**Table 4:** Clinical outcome according to thickness grouping and combined grouping of thickness and pattern

Thickness grouping	CPR	Miscarriage rate	CPR by pattern A^a^	CPR by pattern B^a^	*P*^b^
Group1(n = 52)	23.1%	8.3%	11/45(24.4%)	1/7(14.3%)	NS
Group2(n = 2349)	47.6%	8.9%	1047/2183(48%)	71/166(42.8%)	NS
Group3(n = 495)	55.2%	6.6%	236/432(54.6%)	37/63(58.7%)	NS
*P*^b^	< 0.01	NS	< 0.01	< 0.05	-

Endometrial thickness: Group 1: ≤ 7 mm; Group 2: >7 to 14 mm; Group 3: >14 mm

Pattern A = triple-line pattern, hypoechoic endometrium with well-defined hyperechoic outer walls and central echogenic line

Pattern B = no-triple line pattern, isoechogenic or homogeneous hyperechoic endometrium with a poorly defined or absent central echogenic line

CPR = clinical pregnancy rate

^**a**^Pregnant cycles/total cycles and percentage of clinical pregnancies

^**b**^*P*-values by chi-square text

NS = not significant

### Assessing clinical outcome by combined analysis of endometrial thickness and pattern

To evaluate the relationship between endometrial thickness and pattern, the three endometrial thickness groups were subdivided into the respective endometrial patterns (Table 4). In group 1, pattern A was associated with a clinical pregnancy rate of 24.4%; in pattern B, only 1 cycle obtained clinical pregnancy but ended in miscarriage. Compared with group 2-A, the pregnancy rate was lower in group 2-B, but no significant differences were noted. The early miscarriage rate in group 2-A was significantly lower compared to patterns B (8.3% in pattern A vs 16.9% in pattern B, P < 0.05). In group 3, there was no difference in clinical pregnancy and miscarriage rates between the patterns. Pregnancy rates increased significantly with increasing endometrial thickness in patterns A and B (Table 4).

## Discussion

To our knowledge, this study is the largest in terms of sample size to assess the combined effect of endometrial thickness and pattern on clinical outcome. In our study, we used binary logistic regression to analyze the association between clinical pregnancy and maternal age, endometrial thickness, basal FSH levels, length of stimulation and AFC. We found an independent effect of endometrial thickness on pregnancy rate. This finding is in agreement with previous studies [5,6,14,15]. Pregnancy rate decreased markedly as age increased, in agreement with the results of previous research by others [5,6,14-17].

There is a lack of agreement with regard to the minimum endometrial thickness required for successful pregnancy. In one study, no pregnancies occurred when the endometrial thickness was less than 7 mm [3], whereas other studies have found that a minimum thickness of 6 mm is acceptable as a prerequisite for implantation [18-21], and one study reported a successful pregnancy with an endometrial thickness as little as 4 mm [22]. In the present study, the thinnest endometrial lining for successful ongoing pregnancy was 5.3 mm. From the evaluation of clinical pregnancy rates according to each millimeter of endometrial thickness, we found an endometrial thickness threshold of 7 mm, below which pregnancy rates decreased rapidly. The clinical pregnancy rate in group 1 (endometrial thickness ≤7 mm) was significantly lower than groups 2 and 3, being only 23.1%. The relatively lower pregnancy rate observed in this group suggests that more attention needs to be given to embryos transferred to such patients.

Recently, Richter *et al. *demonstrated a significant increase in the pregnancy rate as endometrial thickness increased, independent of the number and quality of embryos transferred [5]. Their conclusion was confirmed by Ai-Ghamdi *et al *in a 2464-cycle cohort study [6]. Our results were closely similar to these two studies. Although Weissman reported a high miscarriage rate with increased endometrial thickness (>14 mm) [9], in the present study, there was no trend toward an increase in miscarriage rates as endometrial thickness increased from 6 mm to17 mm (see Table 3). Thus, our findings support those of some previous studies in which increased endometrial thickness (>14 mm) did not have a detrimental effect on clinical outcome.

Consistent with several previous studies, we found that endometrial echo patterns have no prognostic value for pregnancy [8,23,24]. However, the miscarriage rate in the no triple-line endometrial pattern was significantly higher than in the triple-line pattern. Several studies have suggested that a premature secretory endometrial pattern is introduced by the advanced P rise, and this premature conversion has an adverse effect on pregnancy rates. In our study, increased P concentrations were not found in no-triple line pattern. The reason that no-triple line endometrial pattern was observed prior to ovulation is not known and cannot be explained by higher P levels. The distribution of the triple-line endometrial pattern in the different endometrial thickness subgroups differed significantly. The exact mechanism for this is not known, and a rational explanation for this phenomenon awaits further study.

In our study, the clinical pregnancy rate was 24.4% in group 1-A, whereas no second trimester pregnancies occurred in group 1-B (see Table 4). Perhaps the coexistence of a thinner endometrium in association with no-triple line pattern reflected a diminished endometrial responsiveness to ovarian hormones and poor receptivity of the endometrium, leading to a low clinical pregnancy rate and poor clinical outcome. Because of the small sample size in group 1-B, further research is needed. Clinical outcomes in pattern B in the other two groups (group 2 and 3) were inconsistent with that in group 1. There were no differences in clinical pregnancy rates between the two patterns in groups 2 and 3. This finding is not in agreement with that of Check *et al*, who found that no pregnancies occurred in those patients with homogeneous hyperechoic endometrium [12]. However, the miscarriage rates in groups 2-B was significantly higher than in group 2-A. These findings suggest that the adverse effect of pattern B together with a moderate endometrial thickness (7-14 mm) mainly affects the pregnancy outcome, but not the pregnancy rate. An unexpected finding was a similar miscarriage rate in the two patterns in group 3. Perhaps adequate endometrial thickness (>14 mm) mitigated the detrimental impact (high miscarriage rate) of pattern B endometrial texture.

One limitation of this study is that the number of thin endometrial thickness (≤7 mm) with no-triple line pattern subjects in the study population was too small to make a definitive statement. The small sample size of no-triple line pattern in each millimeter of endometrial thickness also prevented the use of endometrial thickness as a continuous variable in the combined analysis.

## Conclusions

In conclusion, when a thinner endometrium (≤7 mm) and no triple-line endometrial pattern coexist in an IVF/ICSI candidate, cryopreservation should be recommended. Because endometrial thickness≤7 mm with no-triple line pattern was seen in only 0.3% of cycles in our study, further study is needed to make a definitive conclusion. If a thinner endometrium with a good texture (triple-line) is present, other prognostic factors, such as embryo quality, should be taken into consideration. Regardless of the endometrial pattern, a thicker endometrium (>14 mm) did not have an adverse effect on the clinical outcome. Combined analysis of endometrial thickness and pattern on the day of hCG administration could be more valuable than the separate analyses.

## Competing interests

The authors declare that they have no competing interests.

## Authors' contributions

SLC designed the study, performed the monitoring of endometrium and revised the manuscript. FRW drafted the manuscript and performed the statistical analysis. CL analyzed the data. XC participated in the ultrasound examinations of endometrium. XYS helped to draft the manuscript. HYZ participated in the design of the study. YPN helped to analyze the data. All authors read and approved the final manuscript.
